# Exploring the prognostic value and potential therapeutic strategies of MS4A6A in glioblastoma: A comprehensive analysis of single‐cell and multi‐omics data

**DOI:** 10.1111/jcmm.70177

**Published:** 2024-10-29

**Authors:** Fangchao Wan, Yanling Li, Jianming Zhu, Dandan Yu, Hongjuan Liu, Bohong Hu

**Affiliations:** ^1^ Department of Neurology, Changde Hospital, Xiangya School of Medicine Central South University Changde Hunan China; ^2^ Department of Electrocardiogram, Changde Hospital, Xiangya School of Medicine Central South University Changde Hunan China

**Keywords:** glioma, MS4A6A, multi‐omics, single cell

## Abstract

Glioblastoma (GBM) is a highly aggressive and treatment‐resistant malignancy that poses a significant challenge in modern medicine. Despite advances in surgical resection, radiotherapy and chemotherapy, complete eradication of GBM remains elusive due to its diffuse invasion into the brain parenchyma and propensity for recurrence. The tumour microenvironment (TME), particularly macrophages, has emerged as a critical player in GBM progression, invasion and metastasis. In the immune microenvironment of glioma, MS4A6A exhibits unique expression characteristics in macrophages. This study aimed to investigate the potential role of MS4A6A, a gene associated with aging and neurodegenerative diseases, in GBM and its potential as a prognostic biomarker and therapeutic target.

## 1 INTRODUCTION

Malignant central nervous system tumours are one of the major challenges facing the modern medical field, for which there is no effective treatment.^1^, ^2^ Despite their low incidence, they are among the ten leading causes of cancer‐related deaths. A significant proportion of these primary malignant CNS tumours are malignant gliomas. Glioblastoma (GBM) is the most common malignant glioma in adults and its incidence increases with age.^3^ GBM is a highly malignant tumour that primarily affects the central nervous system and is the most common primary intracranial tumour.^4^, ^5^ With the development of modern medicine, the treatment of GBM mainly includes surgical resection, radiotherapy and chemotherapy, and the integration of multimodal treatments has led to a gradual improvement in the prognosis of patients.^6^ However, complete resection of GBM masses is not possible because the boundaries of GBM masses are often unclear and GBM cells mainly invade the brain parenchyma surrounding the tumour.^7^ In addition, despite advances in therapeutic approaches, the recurrence of GBM is almost inevitable due to the complex aggressive growth behaviour and increasing drug resistance of GBM cells, which can recur rapidly at the primary site or in distant regions of the brain.^8^, ^9^, ^10^ Therefore, there is an urgent need to develop new therapeutic approaches to prolong the survival of GBM patients, reduce recurrence and improve the quality of patient survival.

The tumour microenvironment (TME) is a specialized, complex and highly dynamic mixture of multiple immune cells that is thought to promote tumorigenesis and metastasis.^11^, ^12^, ^13^ Therefore, further understanding of the TME may help to elucidate the underlying mechanisms of GBM development and develop better targeted therapeutic approaches.^14^, ^15^, ^16^ Currently, in the TME, macrophages have attracted extensive attention from researchers.^17^, ^18^, ^19^ This is because macrophages are closely associated with tumour cell proliferation, invasion and metastasis, and these abilities of tumour cells often determine the malignancy of the tumour as well as the prognosis of patients.^20^, ^21^ Macrophages have important functions in immune defence and surveillance.^22^, ^23^ It is a key component of the immune system and excels at performing phagocytosis of pathogens, damaged cells and cancerous lesions, thereby maintaining immune homeostasis and eliminating abnormal cellular components.^24^, ^25^, ^26^ In addition, it has been shown that macrophage activity has a significant prognostic impact on treatment outcomes in cancer patients. Therefore, it is crucial to explore in detail the role of macrophages in GBM, which may lead to new immunotherapeutic strategies.^27^, ^28^, ^29^

Publicly available data from TCGA and GEO enable the study of clinicopathological features of GBM with larger tumour samples, facilitating the screening of GBM biomarkers and the identification of effective drug candidates. The rapid advancement and widespread use of single‐cell RNA sequencing (scRNA‐seq) technology enhance our ability to accurately identify, diagnose, and treat GBM in its early stages. MS4A6A has been linked to aging and the progression of neurodegenerative diseases.^30^ However, the potential biological function of MS4A6A in GBM and its prognostic value for patients with GBM are unknown. In addition, MS4A6A has been shown to be associated with neurodegenerative diseases and disease pathobiology, but whether it is involved in the regulation of GBM and its mechanisms remain unelucidated.^31^, ^32^

Recently, the use of artificial intelligence and machine learning in cancer prognosis research has emerged as a key focus in bioinformatics. These methods have shown significant promise in identifying important genes related to diseases. In this study, we used GBM‐associated single‐cell data to discover signature genes in macrophages and identified one of the key genes, MS4A6A, using multiple machine learning algorithms. We used multi‐omics data to explore the prognostic value, immune profile and potential biological functions of MS4A6A in GBM. More importantly, we screened targeted therapeutic agents against MS4A6A, which contributes to new immunotherapeutic approaches in the future.

## 2 MATERIAL METHODS

### 2.1 Single‐cell data sources and analysis

We downloaded single‐cell sequencing of GBM patient with number GSE162631 from the GEO database. One case of tumour core and one case of paired peripheral tissues were selected for subsequent analysis. Single‐cell correlation analysis was performed using the R package ‘Seurat’. In order to reduce the influence of sequencing depth on the sequencing results, we used the ‘NormalizedData’ function to homogenize the expression matrix. The ‘RunUMAP’ function was used to further downsize the data, and the ‘DimPlot’ function was used for visualization. The ‘FindMarkers’ function was used to analyse the differences between cancer and paraneoplastic tissues.

### 2.2 Clinical significance of MS4A6A

We conducted Receiver Operating Characteristic (ROC) analysis with the pROC package to calculate 95% confidence intervals, the total area under the curve, and smooth ROC curves. This analysis evaluated the diagnostic performance of MS4A6A expression in the tumour disease group compared to the normal group. The calibration curves illustrate how well the fitted model predicts agreement between the tumour group and actual observations. Additionally, the goodness‐of‐fit tests assess whether the observations deviate from the ideal model. We compared the statistical differences in MS4A6A expression between tumour and normal tissues using the GBM dataset.

### 2.3 Biological functional analysis

The 30% of samples with the highest MS4A6A expression were defined as the high expression group, and the 30% of samples with the lowest expression were defined as the low expression group. Difference analysis was performed using the limma package, and gene set enrichment analysis was performed based on the KEGG gene set to calculate the gene set enrichment score ES as well as significance tests and multiple hypothesis tests on the ES values of the gene sets. The 73 KEGG database metabolic gene sets were scored using the GSVA parameter algorithm in R‐package GSVA by integrating the characteristic gene expression to reflect the activity of a given pathway. The *z*‐score algorithm in R package GSVA was implemented for 14 functional state gene sets. Pearson correlation of MS4A6A with each *z*‐score was calculated.

### 2.4 Immunity scores

The easier package was used to compute five immunity scores for TCGA‐GBM. Samples were divided into high and low expression groups according to the median MS4A6A values, and statistical differences in scores between these groups were analysed. Spearman correlation analysis was employed to assess the relationship between MS4A6A and TIP scoring, as well as the autocorrelation within TIP scoring.

### 2.5 Spatial transcriptome data analysis

To accurately assess the cellular composition of each spot on the 10×Visium slides, we applied reverse convolution analysis. This analysis method is based on spatial transcriptomics and single‐cell transcriptomics data. It specifically considers the corresponding cancer type. The scRNA‐seq data were collected from various samples of the same cancer type, leading to the construction of a comprehensive scRNA reference library. To ensure the reliability of the results, we implemented strict quality control measures for single‐cell transcriptome data, focusing on the number of expressed genes, unique molecular identifiers, and the percentage of mitochondrial RNA in each cell. For the screening parameters, we referred to relevant studies on the sources of single‐cell transcriptome data to ensure the scientific validity and accuracy of the screening criteria. Subsequently, by calculating the average expression of the top 25 specifically expressed genes of various cell types in the scRNA‐seq reference for each locus, we constructed a signature score matrix. Finally, we used the get_enrichment_matrix and enrichment_analysis functions in the Cottrazm package to generate an enrichment scoring matrix. This matrix provided strong support for the subsequent cellular composition analysis. The SpatialFeaturePlot function in the Seurat package was used to visualize the enrichment scores of each cell type; the higher the enrichment score, the darker the colour, indicating a higher content of this cell type in the spot.

### 2.6 Machine learning algorithm

The createDataPartition function from the caret package was utilized to randomly split the data into two subsets, with 50% designated as the training set and 50% as the test set. Multiple machine learning models were trained using the train function from the caret package. The explain function from the DALEX package was employed to interpret each model, while the predict function was used to assess model accuracy on the test set and generate ROC curves. The variable_importance function from the DALEX package calculated the importance of variables in the models. Additionally, Lasso regression was performed using the glmnet package.

### 2.7 Immunomodulatory molecules

Immunomodulatory molecules are critical for cancer immunotherapy and many immunomodulatory molecule agonists and antagonists are being evaluated in clinical oncology. We investigated the expression of immunoregulatory molecules and epigenetic control of expression.

### 2.8 Analysis of immune cells

To ensure the quality and consistency of the data, immune infiltration data for all TCGA samples were collected from the publicly available database TIMER 2.0. The Spearman correlation coefficients obtained from the analysis were fully visualized in heatmaps in order to visualize the relationship between different cell types and MS4A6A expression under different algorithms.

### 2.9 Exploration of targeted drugs

To investigate potential therapeutic options to counteract gene‐mediated tumour promotion, we conducted cMAP analysis. We developed a gene‐associated signature comprising the 150 most significantly up‐regulated and 150 most significantly down‐regulated genes by comparing tumours with high and low gene expression. This signature was compared to the cMAP gene signature using the optimal feature matching method XSum (eXtreme Sum) to derive similarity scores for 1288 compounds.

### 2.10 Validation of compound‐target interactions

The crystal structures of the key protein targets were retrieved from the Protein Data Bank (PDB, https://www.rcsb.org/). The 3D structures of the potential active compounds were downloaded from PubChem (https://pubchem.ncbi.nlm.nih.gov/), an open chemical information repository. Molecular docking was conducted, and binding affinities were calculated using AutoDock Vina (http://vina.scripps.edu/).

## 3 RESULTS

### 3.1 Quality control and clustering of GBM single‐cell data

**FIGURE 1:**
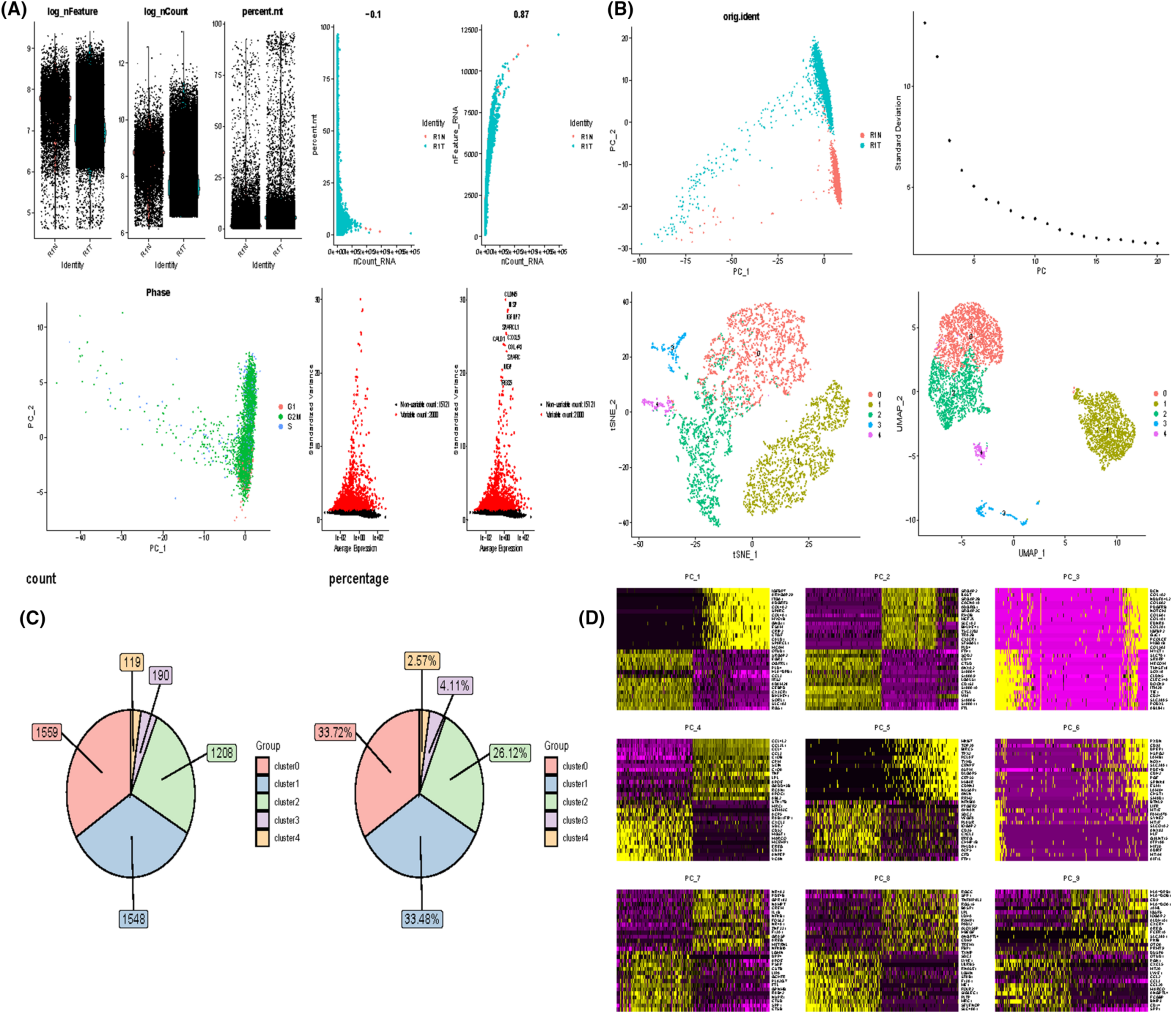
Processing of single cell data. (A) Correlation plots of nFeature_RNA, nCount_RNA, and percent.mt for each cell in the two samples, and nCount_RNA versus percent.mt and nFeature_RNA for each cell. (B) PCA plots of highly variable genes. elbowplot to determine the most appropriate number of principal components. PCA plot of cell cycle genes. Highly variable genes with top ranked standard deviation. UMAP plot after performing dimensionality reduction clustering (C) Cells contained in each cell subpopulation and percentage. (D) Characterized genes in the top nine principal components.

To investigate gene expression at the single‐cell level, we first analysed single‐cell sequencing data from one GBM sample and one paracancerous tissue sample. Figure 1A displays three metrics for the three samples: the number of genes detected in each cell (nFeature_RNA), the total number of mRNA molecules (nCount_RNA), and the proportion of mitochondrial genes (percent.mt). nCount_RNA showed no significant correlation with percent.mt, further indicating that the samples were of good quality for subsequent analysis. We then homogenized the data, searched for cell cycle‐related genes among the highly variable genes, and performed PCA dimensionality reduction on them. The results indicated that the principal component features of cells in the three cell cycles were not fully separated, suggesting that the influence of the cell cycle on subsequent clustering could be disregarded. We found 2000 highly variable genes, with the more significant ones being CLDN5, IBSP, IGFBP7 and others. Additionally, PCA dimensionality reduction using all highly variable genes revealed significant differences between tumour and paracancerous tissues, suggesting that the two are distinctly different in their biological characteristics (Figure 1B). Furthermore, we categorized the cells into five subpopulations. Figure 1C demonstrates the cells contained in each cluster and the percentage of cells, with subgroup 0 having the highest number of cells, accounting for 33.72%. The heatmap illustrates the top 10 characterized genes in the first 9 principal components (Figure 1D).

### 3.2 Characteristic genes of cell subpopulations

**FIGURE 2:**
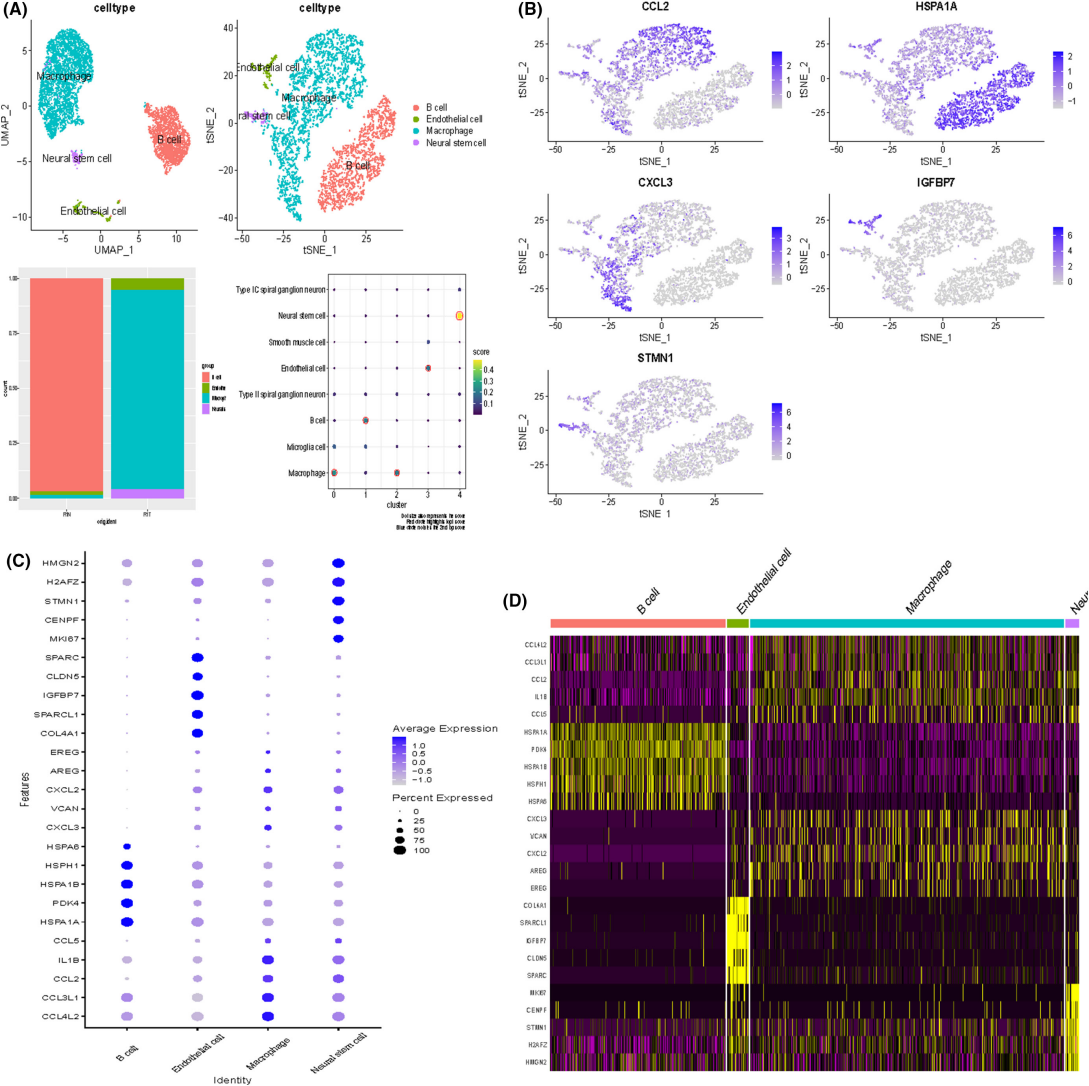
Clustering of single cell data sets. (A) UMAP map after cell annotation. Percentage of cells in cancerous versus paracancerous tissues. Scores of different cell subgroups (B) Characterized gene expression. (C) Bubble plots of marker gene expression for each cell population. (D) Heatmap of marker gene expression for each cell population.

Figure 2A demonstrates the 2D spatial distribution of single‐cell transcriptome data from two patient screens after downscaling by t‐SNE and UMAP data. To determine the cell types of the five subpopulations, we scored the different subpopulations, with cluster0 and 2 macrophages having the highest scores, cluster1 being B cells, cluster3 being epithelial cells, and cluster4 being neural stem cells. Also in R1N patients, the highest percentage of B cells was found, while in R1T patients, the highest percentage of macrophages was found. The characteristic gene expression of each subpopulation is shown in Figure 2B. ccl2 and cxcl3 were mainly expressed in macrophage, while HSPA1A was mainly expressed in B cells. Bubble plots and heat maps demonstrated the expression of marker genes in different cell types labeling each cell population (Figure 2C,D).

### 3.3 Machine learning screening of key genes in macrophages

**FIGURE 3:**
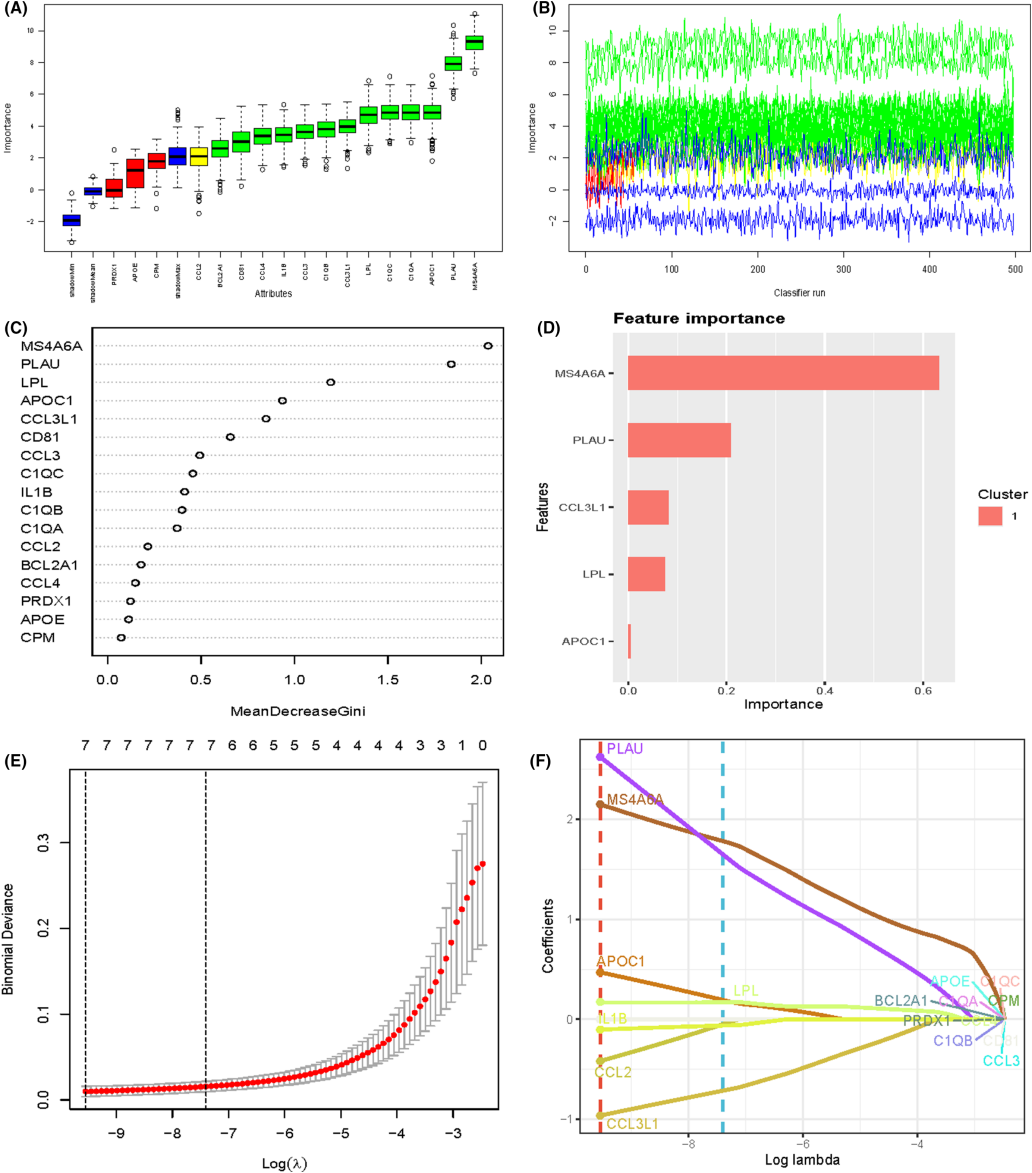
Machine learning screening of key genes in macrophages. (A) boxplot showing the distribution of a variable (possibly expression levels) across different categories or samples. (B) A line plot indicating the variation of a variable over time or across different samples. The colours suggest multiple groups or clusters. (C) A plot showing feature importance or contribution, possibly from a machine learning model. The *x*‐axis represents the mean decrease in accuracy or Gini index, suggesting the importance of each feature. (D) A bar plot of feature importance, highlighting the most significant features in a specific cluster.(E) A plot showing cross‐validation results for a model, likely a regularization path for LASSO regression. (F) A coefficient path plot.

We performed an in‐depth analysis of the top 20 genes expressed in macrophages in an attempt to find the key. We screened these genes using multiple machine learning algorithms. Interestingly, MS4A6A was in the key in multiple machine learning algorithms. We therefore performed a follow‐up analysis of it (Figure 3A–F).

### 3.4 Comparative analysis of different machine learning models on residual distributions

**FIGURE 4:**
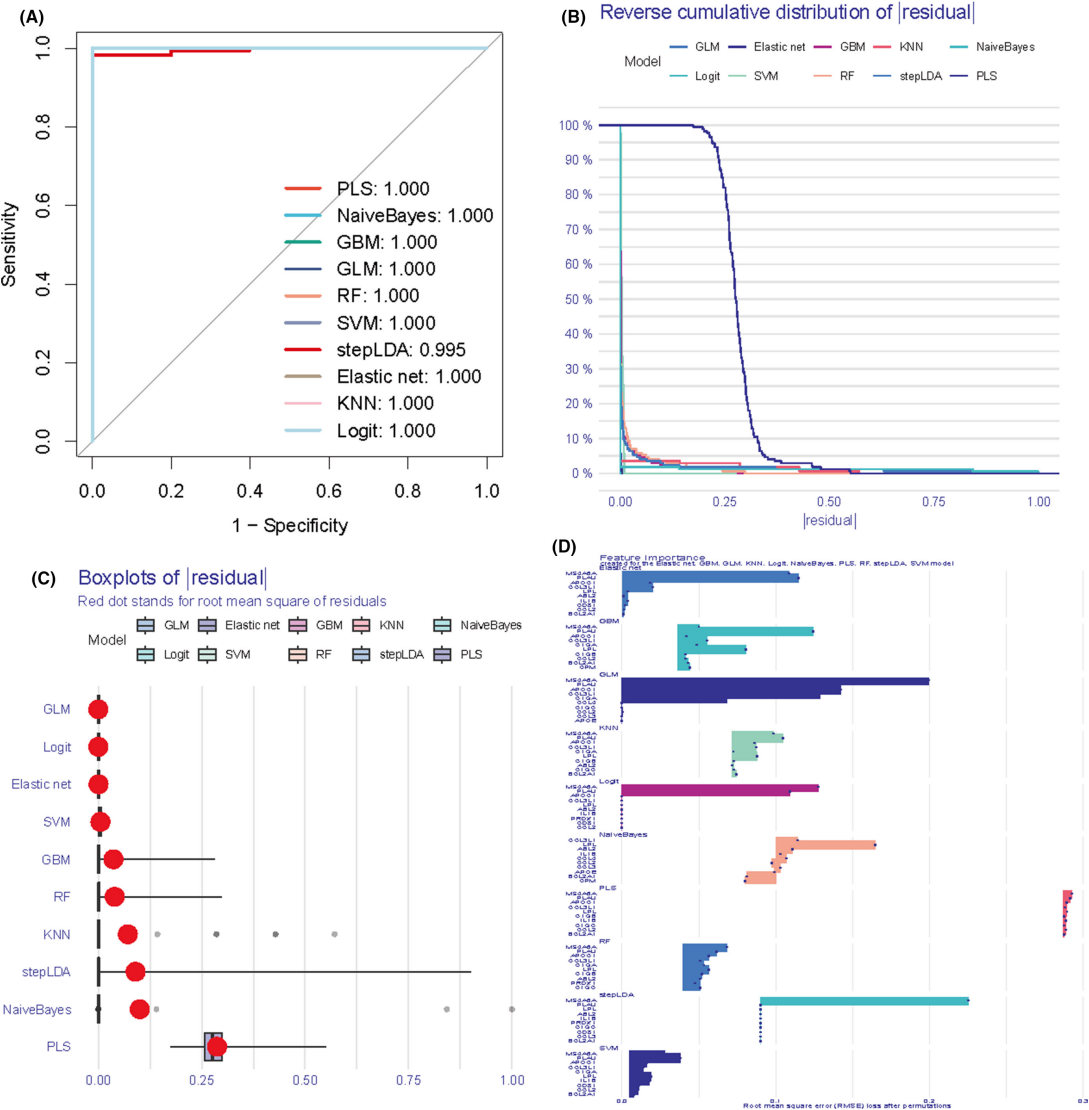
Evaluation of different machine learning. (A) ROC curves to assess binary classifier performance. the closer the ROC curve is to the upper left corner, the better the performance of the model. (B) Inverse cumulative distribution plot of residuals for the sample. (C) Residual boxplot showing the median, interquartile range and outliers of the residuals. (D) Top ten genes for each model importance.

We provide an in‐depth analysis of the performance of a range of machine learning models on specific datasets, with a particular focus on their residual distribution properties. By comparing the inverse cumulative distribution function and boxplots of different models, we aim to assess the predictive accuracy and generalization ability of the models. In this study, we chose models including Generalized Linear Model (GLM), Elastic net regression (Elastic net), Gradient Boosting Machine (GBM), K Nearest Neighbours (KNN), NaiveBayes, Logistic Regression (Logit), Support Vector Machines (SVM), Random Forests (RF), Stepwise Linear Discriminant Analysis (stepLDA), and Partial Least Squares Regression (PLS) were compared (Figure 4). The results show that all the models exhibit a high degree of consistency at the extremes of the residual distributions, with PLS, NaiveBayes, GBM, GLM, RF and SVM reaching 100% at the cumulative percentage of 1.0, indicating that the prediction errors of these models are small at the extremes. The box plots further reveal the distribution of the residuals of each model, where the red dots represent the root mean square of residuals (RMSR), providing us with a visualization of the prediction error of the models. In all of these models, MS4A6A is in an important position.

### 3.5 MS4A6A expression differences and clinical significance

**FIGURE 5:**
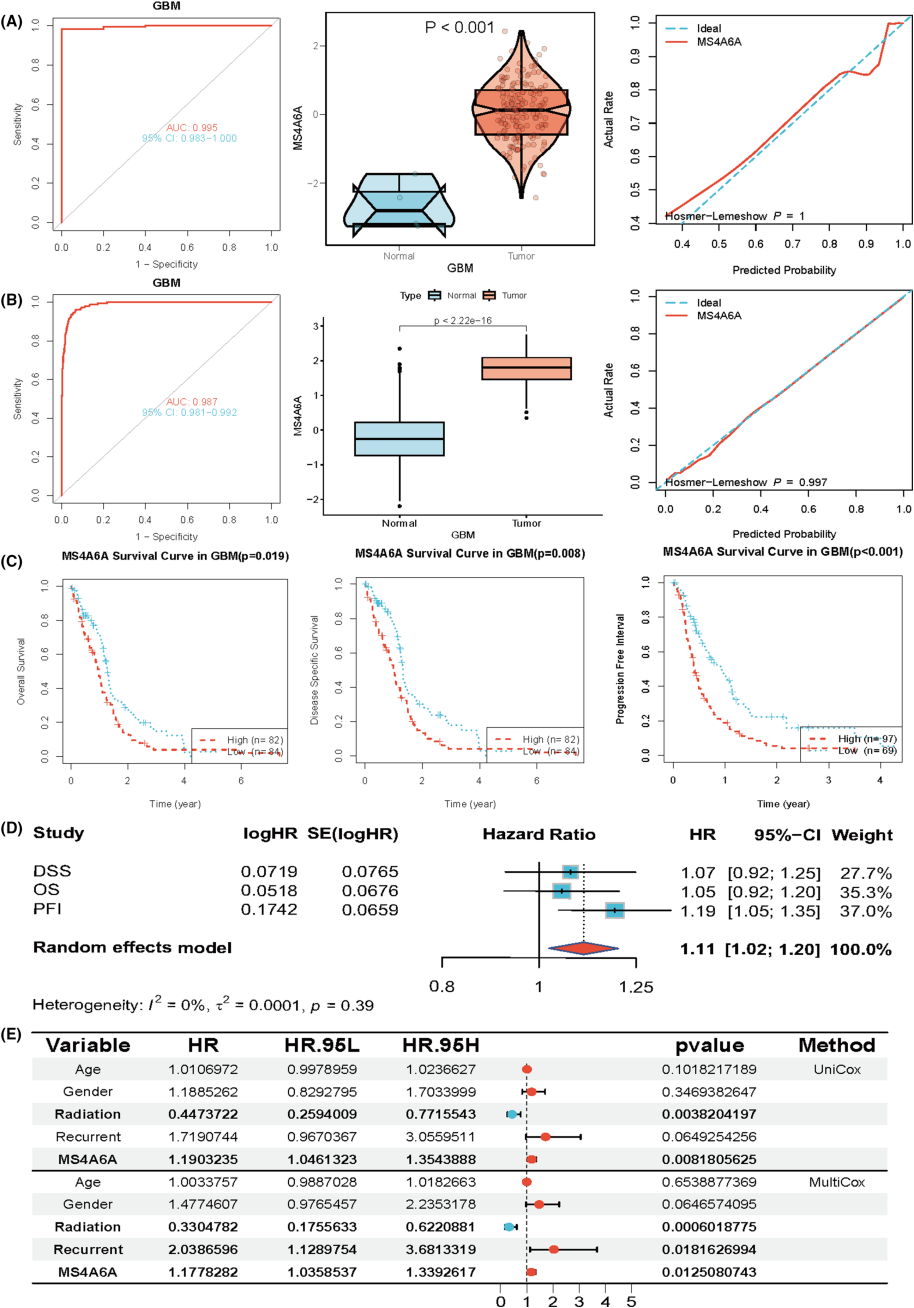
Clinical significance of MS4A6A. (A, B) Data from TCGA‐GBM and TCGA‐GBM combined with GTEx were analysed. (C) The prognostic value of MS4A6A. (D, E) The forest plot demonstrates the specific effect of MS4A6A on survival time in three unifactorial and multifactorial analyses.

To determine whether MS4A6A expression is elevated in tumours and its clinical significance for GBM, we analysed data from TCGA‐GBM and TCGA‐GBM combined with GTEx, respectively. The results showed that MS4A6A had an extremely strong diagnostic value, with AUCs of 0.995 and 0.987 in the two datasets, respectively (Figure 5A,B). The expression of MS4A6A in GBM tissues was much higher than that in normal tissues. In addition, the calibration curves and goodness‐of‐fit tests of the predictions of the tumour group and the normal group indicated that MS4A6A was a predictor of diagnosis of GBM, and no deviation from a perfect fit was detected, implying that the prediction of whether it was a tumour tissue or not by using MS4A6A was not significantly different from the ideal model. The KM curves indicated that the patients with high expression of MS4A6A had a shorter OS, DSS and PFI (Figure 5C). The forest plot demonstrated the specific effects of MS4A6A on the three survival times (Figure 5D). In addition, unifactorial and multifactorial also showed that MS4A6A could be used as a prognostic indicator for GBM (Figure 5E).

### 3.6 Potential biological functions of MS4A6A

**FIGURE 6:**
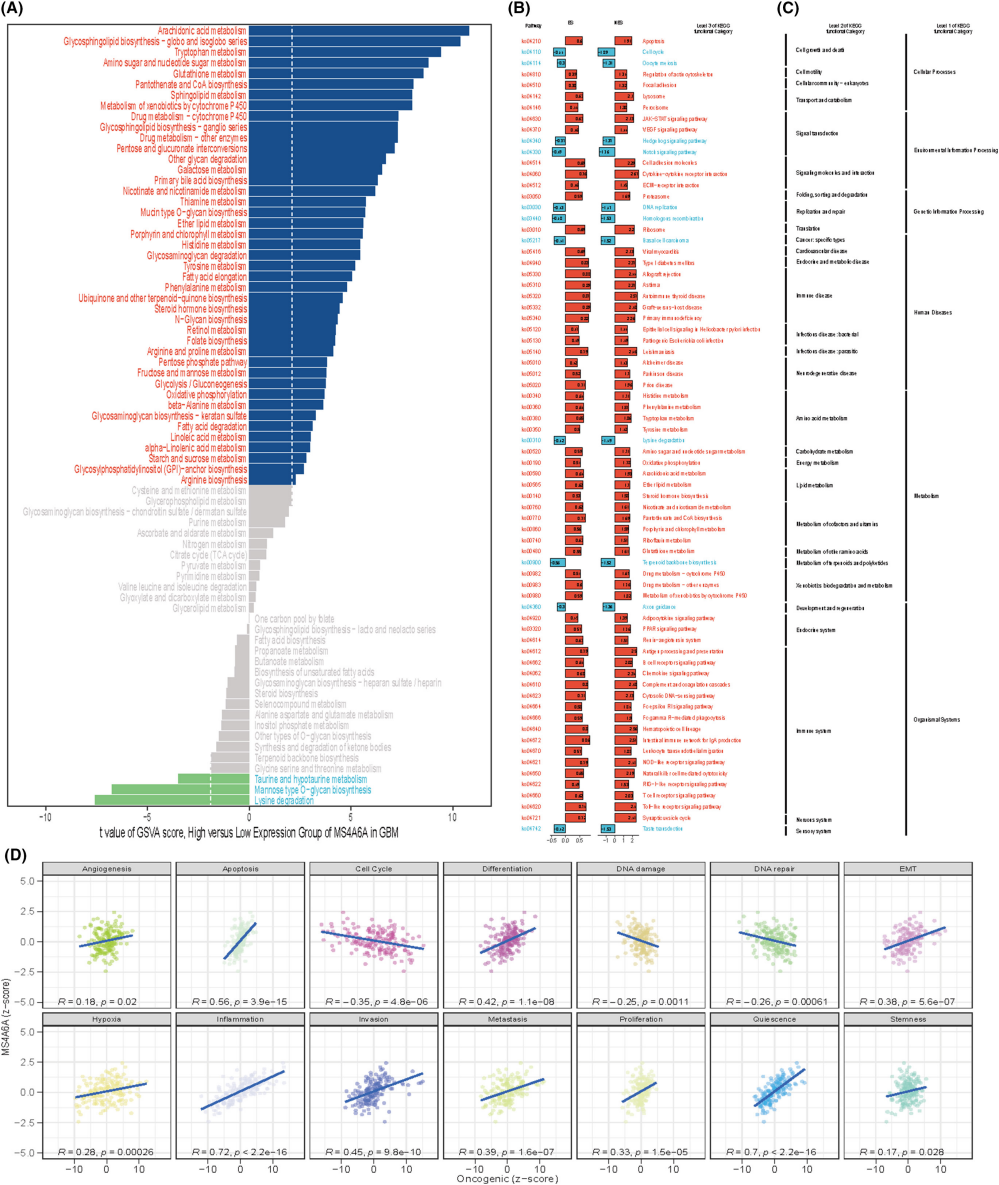
Biological function of MS4A6A. (A) Differences in metabolic gene sets between the high and low groups. *x*‐axis is the *t*‐value of GSVA scores. (B) Enrichment analysis results for the high and low expression groups. (C) Horizontal coordinates are scores for each functional state, (D) vertical coordinates are MS4A6A expression and R is pearson correlation analysis.

To elucidate the role of MS4A6A in GBM, we conducted a detailed analysis of its biological functions. Using the GSVA package in R, we applied the GSVA parameter algorithm to score a set of 73 KEGG metabolic genes. Samples with the top 30% MS4A6A expression were classified as the high expression group, while those with the lowest 30% were classified as the low expression group. The limma package was used to compare GSVA scores of metabolic gene sets between these groups. The analysis revealed that the high expression group showed activation in arachidonic acid metabolism and significant inhibition in lysine degradation (Figure 6A). Additionally, gene set enrichment analysis based on KEGG pathways indicated that the high MS4A6A expression group was enriched in apoptosis, T cell receptor signalling, prion disease, and other pathways, whereas the low expression group was enriched in DNA replication, Notch signalling, cell cycle and more (Figure 6B). Furthermore, we calculated the *z*‐scores of 14 tumour states using GSVA and assessed the Pearson correlation between MS4A6A expression and GSVA scores. The results demonstrated a significant positive correlation of MS4A6A expression with DIFFERENCE and APOPTOSIS, aligning closely with the enrichment analysis findings (Figure 6C).

### 3.7 Relationship between MS4A6A and immunization scores

**FIGURE 7:**
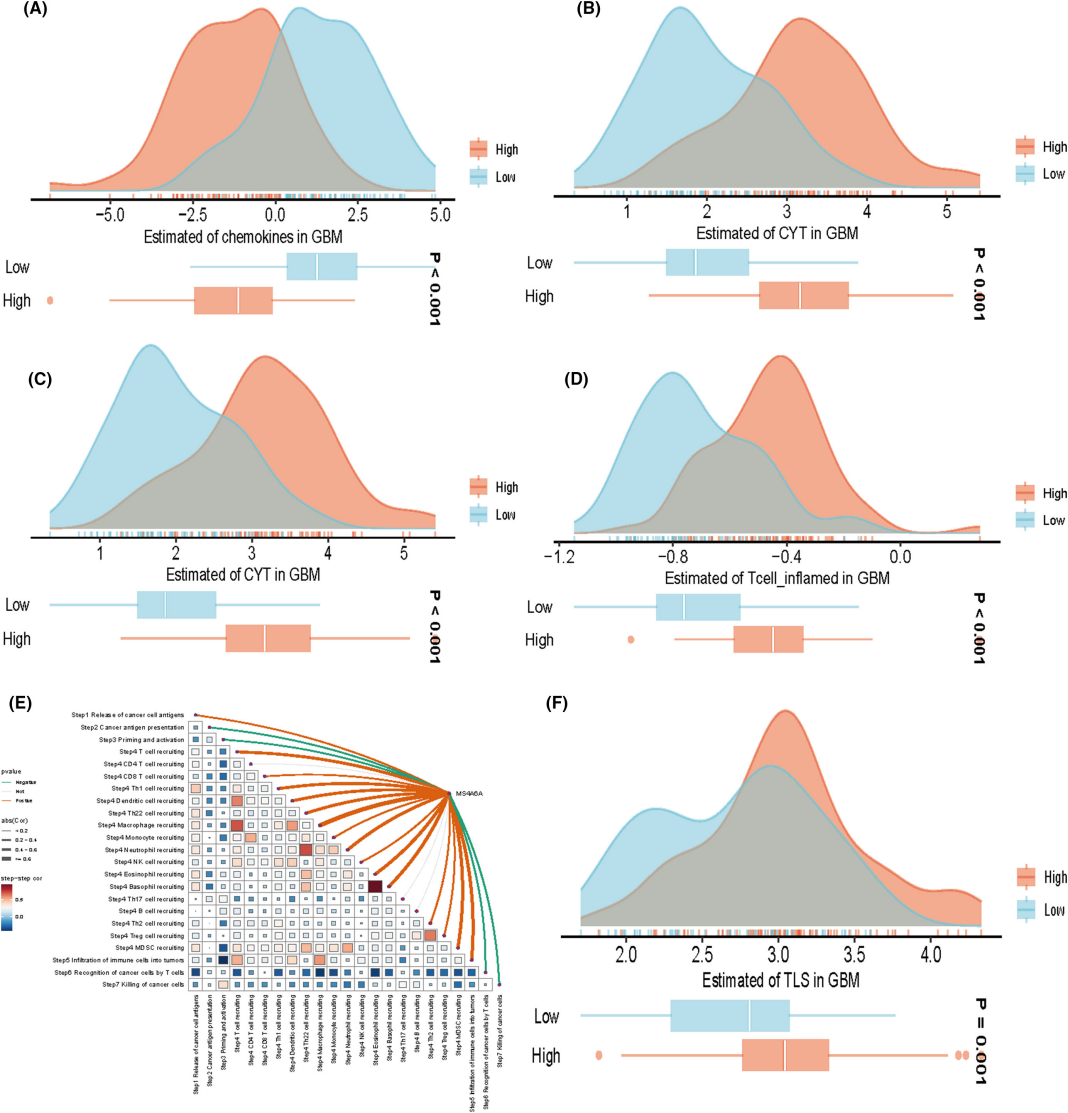
Association of specific gene expression with immune infiltration and genomic status based on scoring for characterizing immunogenicity, DNA damage, etc. spearman correlation between TIP scoring and MS4A6A expression. Five scoring differences in the MS4A6A high/low expression group (A–D, F). The upper panel provides the distribution of scoring levels across samples in the MS4A6A high/low expression group. The ends of the lower box indicate the interquartile range of values. The line in the box indicates the median value (E).

Based on the quartiles of MS4A6A expression, all patients were categorized into four types, that is, Q1, Q2, Q3 and Q4, with Q1 representing the 25% of samples with the highest MS4A6A expression and Q4 representing the 25% of samples with the lowest MS4A6A expression. Based on the results of previous studies of immune response and genomic status, we calculated the average of each scoring across the four patient types. The heatmap represents, from left to right, the within‐group averages of the immune response scores versus genomic status scores for each of the Q1, Q2, Q3, Q4 subtypes, with the Q1 group with high MS4A6A expression also having higher scores. We sought to analyse the relationship between MS4A6A and the anti‐cancer immune status of the seven‐step cancer immune cycle. Specifically, these include cancer cell antigen release, cancer antigen presentation, initiation and activation, immune cell transport to the tumour, immune cell infiltration into the tumour, and T cell recognition of cancer cells and killing of cancer cells. TIP (Tracking Tumour Immunophenotype) was used to quantify the scoring for each tumour, at each step. The results showed a significant positive correlation between MS4A6A and these immune states. easier is a tool for predicting biomarker‐based immunotherapies based on a model of cancer‐specific immune responses, with the goal of predicting anti‐tumour immune responses from RNA‐seq data. We used TCGA‐GBM data to calculate the differences in five scores, cytolytic activity, tertiary lymphoid structure, interferon‐γ signature, inflammatory T cells and chemokines, in the MS4A6A high/low expression group. The results showed that all four scores, except for the chemokine score, were higher in the high expression group (Figure 7A–F).

### 3.8 Relationship between MS4A6A and immune cells

**FIGURE 8:**
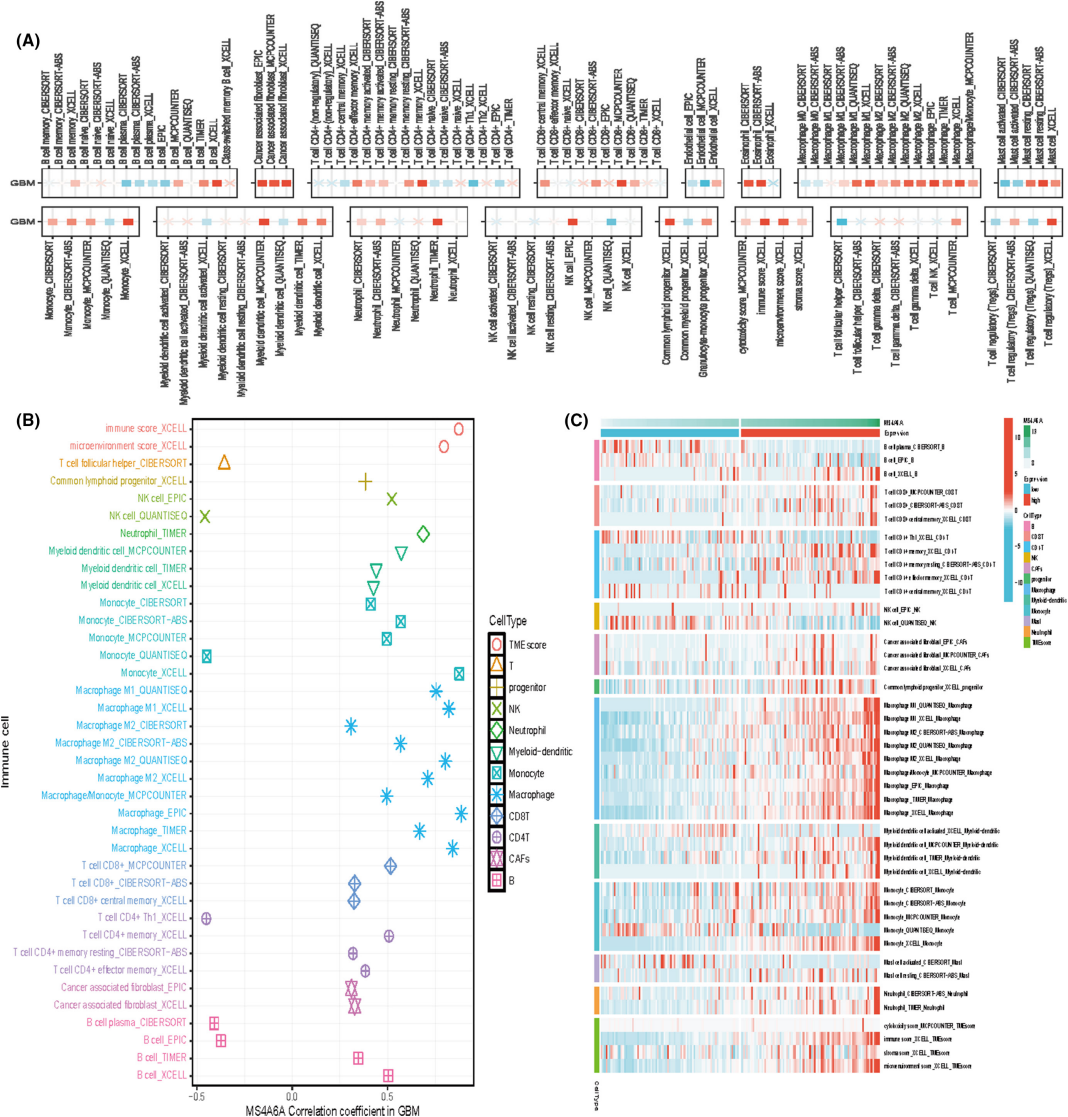
Relationship between MS4A6A and immune cells (A, B) Multiple algorithms to assess the Spearman correlation of MS4A6A expression with different immune‐infiltrating cells in GBM. The colour of the squares reflects the correlation coefficient (*p* < 0.05), with the redder colour representing the value closer to 1 (positive correlation) and the bluer colour representing the value closer to −1 (negative correlation). (C) Immune cell infiltration in the high and low expression groups.

A thorough analysis of various algorithms can enhance our understanding of the mechanisms and characteristics of immune infiltration, thereby shedding light on tumour pathogenesis and offering new approaches for disease diagnosis and treatment. Therefore, we aimed to understand the relationship between MS4A6A and immune cells in GBM. We used a multi‐algorithm approach to assess the Spearman correlation of MS4A6A expression with different immune infiltrating cells. The results indicated a positive correlation between MS4A6A and most immune cells, with tumour‐associated fibroblasts and M1 and M2 macrophages showing particularly strong associations. The heat map indicated greater immune cell infiltration in the group with high MS4A6A expression (Figure 8A–C).

### 3.9 Relationship between MS4A6A and immunomodulators

**FIGURE 9:**
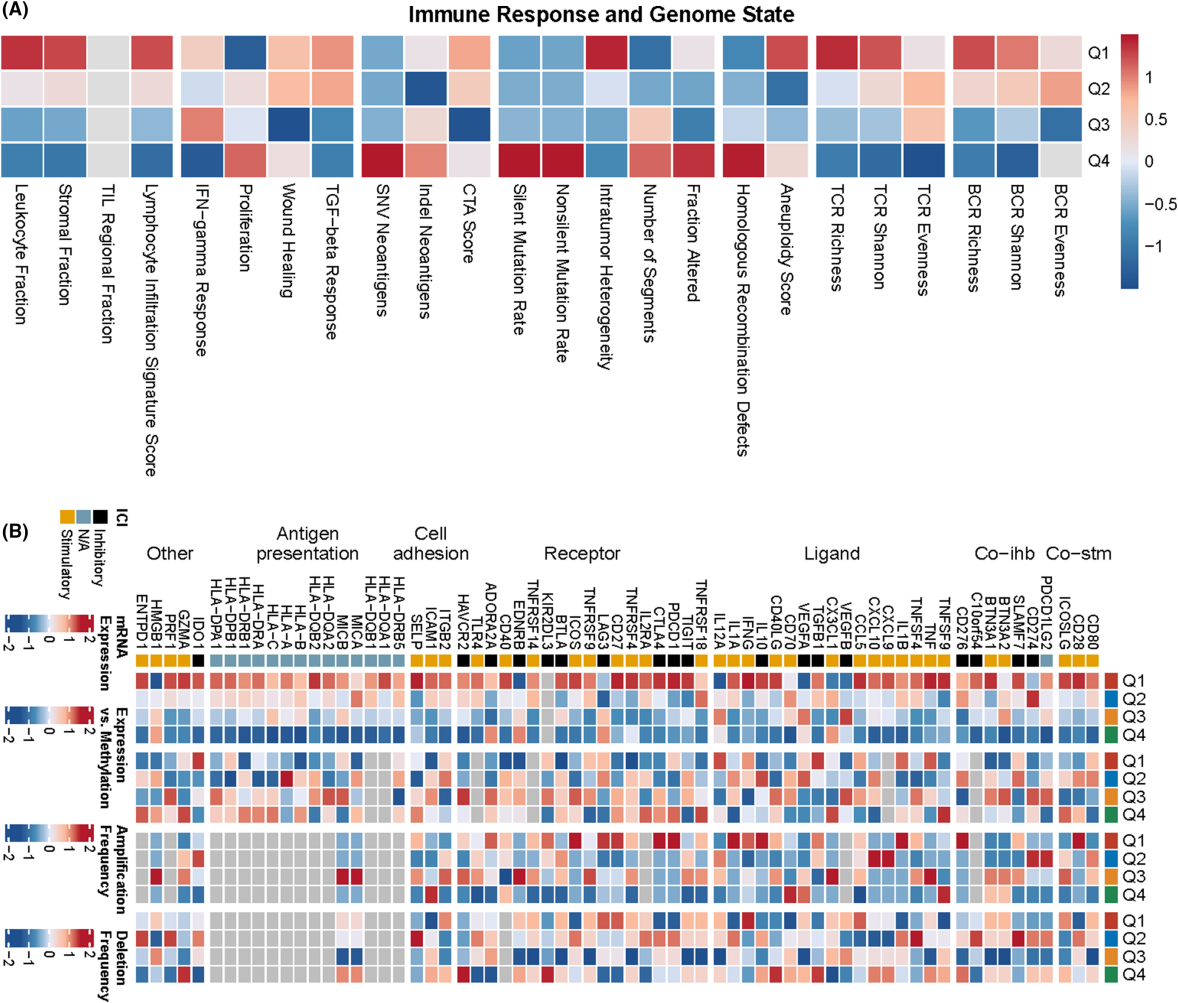
Heatmap representing from left to right the within‐group mean of each immune response score versus genomic status score for subtypes Q1, Q2, Q3, Q4, normalized by rows thus allowing all scores to be scaled to the same range(A). mRNA expression is the median of the normalized expression levels. Expression versus methylation is the correlation between gene expression and dna methylation *β* values. Amplification frequency is the difference between the proportion of samples amplified by immunoregulators in a particular isoform and the proportion amplified in all samples. Absence frequency is the difference between the proportion of samples in which immunoregulators are absent in a particular isoform and the proportion of samples in which they are absent in all samples (B).

Based on the quartiles of MS4A6A expression, all patients were categorized into four types, that is, Q1, Q2, Q3 and Q4 (Q1 represents the 25% of samples with the highest expression of MS4A6A, and Q4 represents the 25% of samples with the lowest expression). We explored the association of MS4A6A gene expression with immune infiltration and genomic status based on scoring for characterizing immunogenicity, DNA damage, etc (Figure 9A). Further, we explored the relationship between MS4A6A and immunomodulators. Thereby, we understood their expression and control patterns in different states of MS4A6A (Figure 9B). All these results suggest that MS4A6A plays an important role in the regulation of the immune system.

### 3.10 Exploring the association of MS4A6A with immune cells using spatial transcriptome data

**FIGURE 10:**
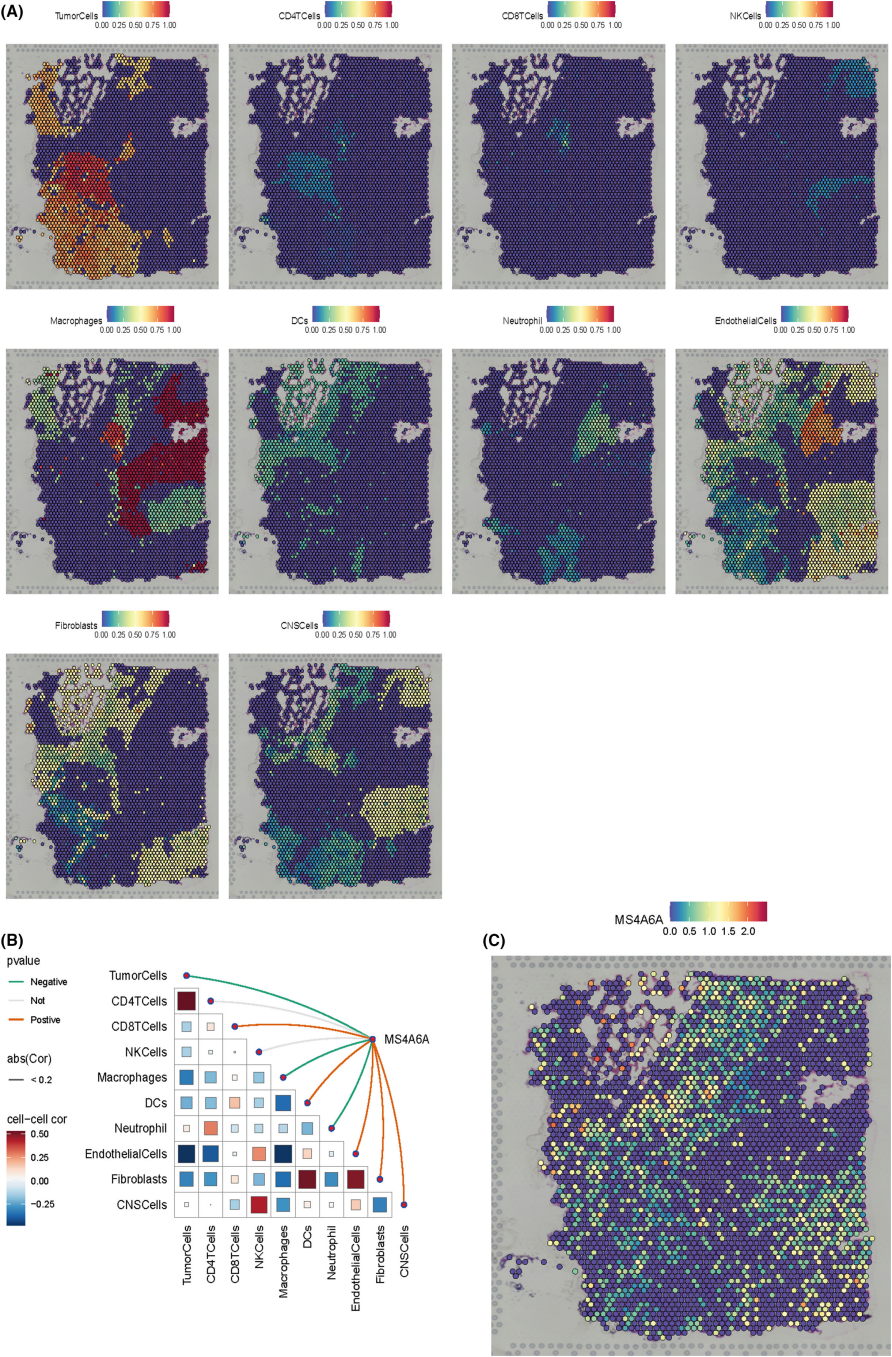
Spatial transcriptomic analysis. (A)MS4A6A expression in various immune cells such as Tumour Cells, CD4 T Cells, CD8 T Cells, NK Cells, Macrophages, DCs, Neutrophils, Endothelial Cells, Fibroblasts and CNS Cells. Each dot is a microregion (spot) of spatial transcriptome sequencing, darker colour (red) means higher expression of that gene in the spot. (B) Spearman correlation analysis was performed to calculate the correlation between cellular content and cellular content, as well as the correlation between cellular content and gene expression in all spots. Red lines represent positive correlations, green lines represent negative correlations, grey lines represent no significance and the thickness of the line represents the absolute magnitude of the correlation coefficient. The correlation of the triangular region is indicated by the colour shade and size of the squares. (C) Expression of MS4A6A.

To further validate these results, we utilized spatial transcriptome data to further analyse the potential association of MS4A6A with immune cells. Based on the deconvolution results, we calculated the cell type with the highest content in each microregion and visualized the cellular component maxima in each microregion using the SpatialDimPlot function in the Seurat package (Figure 10A). We also visualized the MS4A6A expression landscape in each microregion (Figure 10C). Meanwhile, spearman correlation analysis was used to calculate the correlation between cellular content and cellular content, and between cellular content and MS4A6A expression in all SPOTs (Figure 10B). The results showed that MS4A6A had a significant positive correlation with CNS cells, fibroblasts and CD8T cells.

### 3.11 Targeted drugs for MS4A6A

**FIGURE 11:**
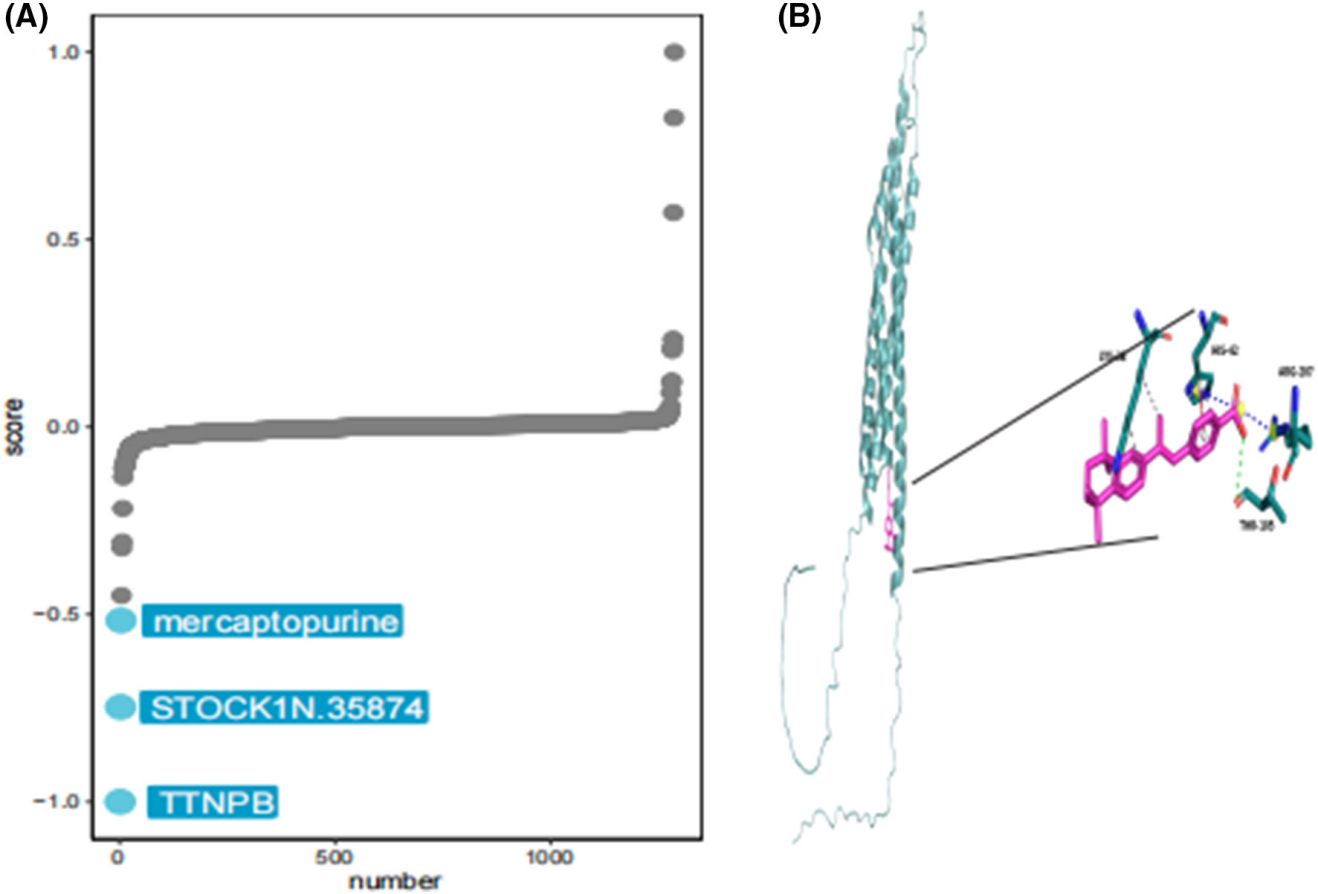
Target drug for MS4A6A. (A) cMAP analysis to explore potential therapeutic options that can counteract MS4A6A‐mediated tumour promotion. (B) Molecular docking indicates that TTNPB and MS4A6A can bind efficiently.

To investigate therapeutic options to counteract MS4A6A‐mediated tumour promotion, we conducted cMAP analysis. We developed a gene signature consisting of the 150 most significantly up‐regulated and 150 most significantly down‐regulated genes by comparing tumours with high and low gene expression. This signature was compared to the cMAP gene signature using the XSum (eXtreme Sum) method to obtain similarity scores for all compounds. The results indicate that TTNPB may reverse the molecular signature associated with MS4A6A dysregulation, counteracting its pro‐cancer effects. Molecular docking showed strong binding interactions between TTNPB and MS4A6A, with a reliable binding energy of −6.9 kcal/mol. The conformations of TTNPB and MS4A6A are illustrated in the Figure 11A and 11B.

### 3.12 Association of MS4A6A with immune cells

**FIGURE 12:**
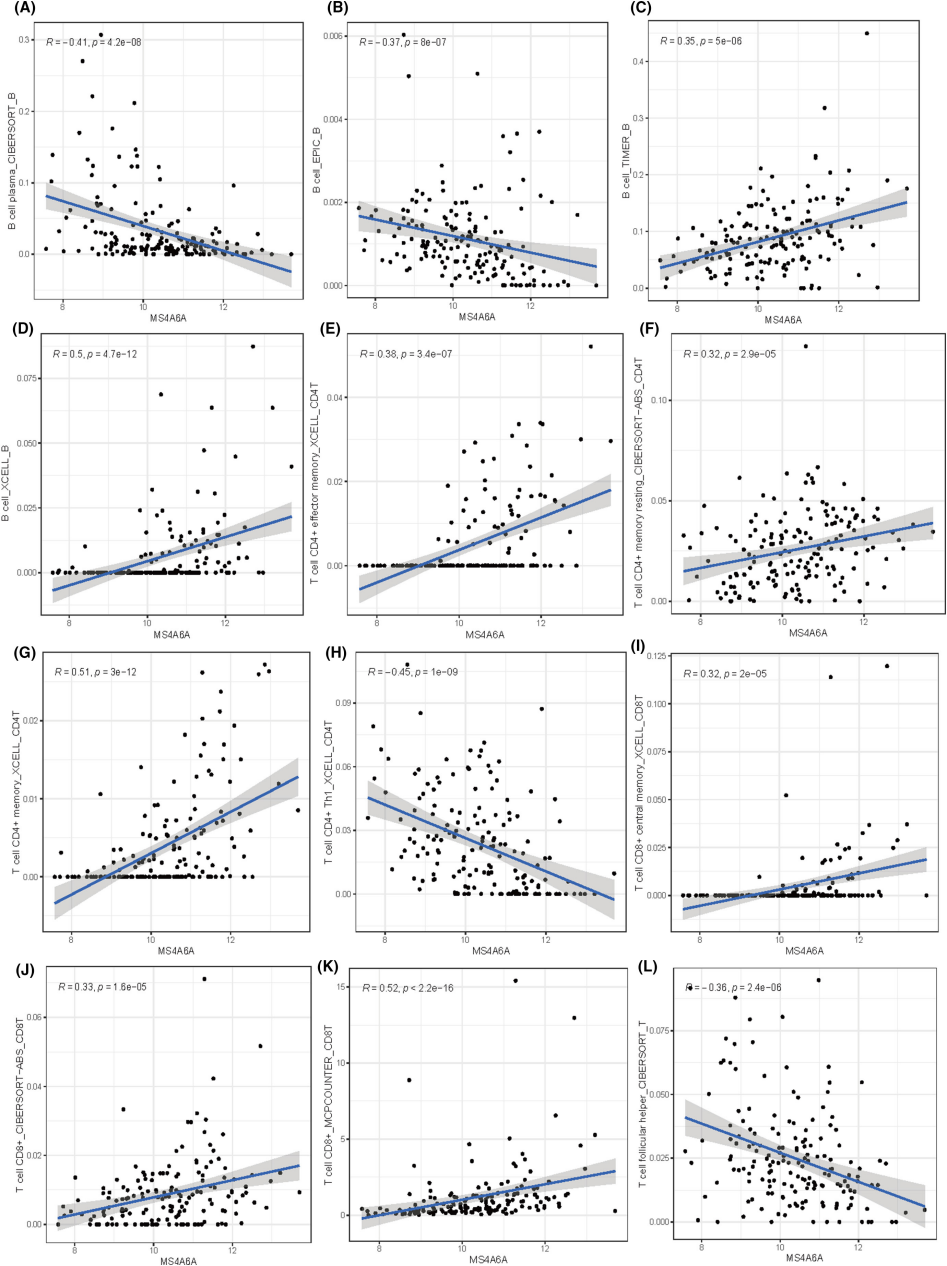
Correlation between MS4A6A and immune cells. (A–L) Correlation of MS4A6A with multiple types of immune cells. (A) B cell plasma_CIBERSORT_B. (B) B cell_EPIC_B. (C) B cell_TIMER_B. (D) B cell_XCELL_B. (E) T cell CD4+ effector memory_XCELL_CD4T. (F) T cell CD4+ memory resting_CIBERSORT−ABS_CD4T. (G) T cell CD4+ memory_XCELL_CD4T. (H) T cell CD4+ Th1_XCELL_CD4T. (I) T cell CD8+ central memory_XCELL_CD8T. (J) T cell CD8 + _CIBERSORT−ABS_CD8T. (K) T cell CD8 + _MCPCOUNTER_CD8T. (L) T cell follicular helper_CIBERSORT_T.

We calculated the correlation between MS4A6A and immune cells using various algorithms. The results showed that MS4A6A was significantly positively correlated with CD8T cells in multiple algorithms (Figures 11, 12).

### 3.13 Differential expression of immune‐related molecules in MS4A6A high/low expression groups

**FIGURE 13:**
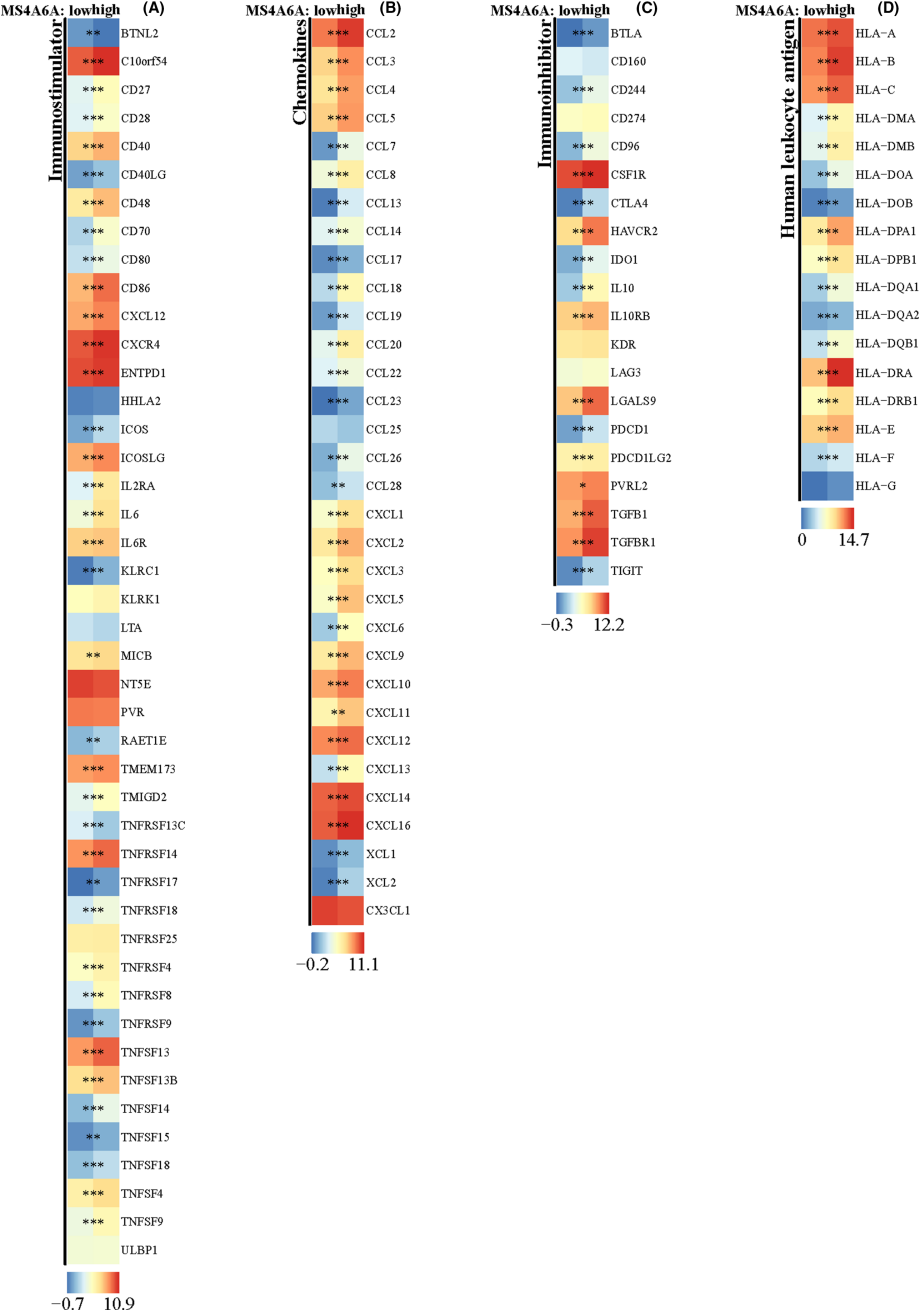
Differences of immune‐related genes in high/low MS4A6A groups. (A–D) Expression differences of immunostimulatory genes (A), chemokine‐related genes (B), immunosuppressive genes (C) and human leukocyte antigen‐related genes (D) in MS4A6A high/low expression groups. *p<0.05, **p<0.01, ***p<0.001.

To understand the further relationship between immune‐related molecules and MS4A6A, we explored the expression differences of four types of genes (immune‐stimulating genes, immune‐suppressing genes, chemokines and human leukocyte antigens) in the high and low expression groups of MS4A6A. The results showed that most genes were more highly expressed in the MS4A6A high expression group (Figure 13).

## 4 DISCUSSION

Although there have been advances in therapeutic strategies for gliomas over the past few decades, the median overall survival after diagnosis remains around 15 months, indicating unsatisfactory long‐term outcomes.^33^ Given the poor prognosis for glioma patients, we urgently need to identify new biomarkers or molecular targets to enhance the diagnosis, prognosis and treatment of glioma. This study aims to explore new and effective targets for personalized therapeutic management and treatment of glioma. Macrophages play a crucial role in cancer by promoting tumour growth, metastasis and the formation of new blood vessels.

Currently, machine learning based on big data has made rapid progress in the biomedical field and achieved promising results.^34^ Clinical big data refers to large and diverse data originating from complex sources.^35^, ^36^ Machine learning refers to a discipline that studies algorithms focused on finding a pattern in large‐scale data and using those patterns to make predictions. Using machine learning methods, researchers can analyse a variety of histological data, including gene expression, RNA sequences, non‐coding RNAs (e.g. miRNAs) and protein expression and modifications. We can classify different cancers into more detailed subtypes to predict cancer progression and assess treatment response. More importantly, the combination of clinical big data and machine learning can help us find key variables among the many influencing factors.^37^, ^38^, ^39^

In our study, based on multi‐omics and multiple machine learning approaches, we not only found that MS4A6 is a key gene for macrophages in GBM, but also found that it accurately predicts poor prognosis in gliomas, correlating with the biologically malignant characteristics of numerous tumours. In addition, cMAP analysis and molecular docking showed that TTNPB and MS4A6A could bind efficiently. These results reveal that MS4A6A may be involved in macrophage infiltration in GBM and influence the outcome of immunotherapy.

In our study, we used public cancer databases to analyse data and discovered that MS4A6A is highly expressed in glioma tissues compared to normal tissues. We analysed the survival of GBM patients across multiple datasets and found that MS4A6A serves as a poor prognostic factor while also demonstrating good diagnostic efficacy. We explored the potential biological functions of MS4A6A through enrichment analysis and GSVA scoring. The results indicate that MS4A6A may play a role in activating arachidonic acid metabolism while simultaneously inhibiting lysine degradation. Additionally, our findings from various algorithms showed that MS4A6A is strongly associated with apoptosis, the T cell receptor signalling pathway, prion disease and other pathways.

In recent years, it has become clear that immune cells can either suppress tumours or support their growth. However, the relationship between MS4A6A and immune infiltration in gliomas has not been studied. Our research demonstrated that MS4A6A is positively correlated with most immune cells, particularly tumour‐associated fibroblasts and M1 and M2 macrophages. GBM patients with high MS4A6A expression also showed higher levels of immune cell infiltration. This suggests that MS4A6A may negatively impact patient prognosis by promoting malignant behaviour and immune cell infiltration. Our findings indicate that MS4A6A expression could serve as an indicator for identifying patients who might benefit from anticancer immunotherapy.

The limitation of this study lies in its reliance on existing public databases, such as TCGA and CGGA. While these databases provide a large amount of data, they may suffer from selection bias or data incompleteness. Despite the use of multiple omics and machine learning methods, bioinformatics analysis may not fully capture the complexity of all biological processes and molecular mechanisms. The immune microenvironment of gliomas is highly complex, involving multiple cell types and signalling pathways. Although studies have indicated the association of MS4A6A with immune cell infiltration, there may be other unknown factors affecting immune response and treatment outcomes.

## 5 CONCLUSION

In summary, we identified MS4A6A using single‐cell data as well as machine learning algorithms. MS4A6A plays an important role in prognostic, immune and biological functions in gliomas.

## AUTHOR CONTRIBUTIONS

**Fangchao Wan:** Conceptualization (equal); data curation (equal); writing – original draft (equal). **Yanling Li:** Data curation (equal); formal analysis (equal). **Jianming Zhu:** Data curation (equal); funding acquisition (equal). **Dandan Yu:** Formal analysis (equal); methodology (equal). **Hongjuan Liu:** Investigation (equal). **Bohong Hu:** Investigation (equal); supervision (equal); writing – review and editing (equal).

## CONFLICT OF INTEREST STATEMENT

The authors have no conflicts of interest to declare.

## Data Availability

Data are available from the corresponding author.
